# Immunological and Pathogenic Differences of Two Experimental Bluetongue Virus Serotype Infections Evaluated in Two Disparate Host Species

**DOI:** 10.3390/v16101593

**Published:** 2024-10-10

**Authors:** Joseph A. Westrich, Erin E. McNulty, Madison Stoltz, Tyler J. Sherman, Molly Carpenter, Mollie Burton, Amy Nalls, Hennio S. Rubio, Audrey Sandoval, Christie Mayo, Candace K. Mathiason

**Affiliations:** Department of Microbiology, Immunology, and Pathology, Colorado State University, Fort Collins, CO 80523, USA; joseph.westrich@colostate.edu (J.A.W.); tyler.sherman@colostate.edu (T.J.S.); amy.nalls@colostate.edu (A.N.);

**Keywords:** bluetongue virus, immune response, BTV10, BTV17, sheep, muntjac deer

## Abstract

Bluetongue virus (BTV) is a prevalent midge-borne pathogen that infects ruminant species worldwide. BTV infections range from asymptomatic to lethal, with mechanisms that determine the severity of infection remaining largely undefined. Although it is relatively poorly understood, the immune response to BTV infection is thought to be critical for both the propagation of disease as well as the resolution of infection. To bridge this gap in knowledge, we infected cohorts of sheep and muntjac deer with two serotypes of BTV (BTV10 and BTV17) for longitudinal analysis (30 days). Interestingly, species-specific differences were observed. Circulating virus was detected early and remained detectable for the duration of the sheep study, while infections in muntjac showed faltering detection of BTV10 at 3 weeks post infection. The magnitude of the immune response was subdued in the muntjac when compared to the sheep cohorts, though similar responses were observed. We also assessed midge viral uptake and the ability to replicate BTV. Midges successfully fed on both species, yet those that fed on sheep resulted in more efficient BTV transmission. Our findings demonstrate that differences in BTV infections, immune responses, and vector competence across host species and serotypes will impact global BTV emergence and strategies for mitigation.

## 1. Introduction

Bluetongue (BT) disease is an insect-borne viral infection of ruminants and is caused by bluetongue virus (BTV). It is principally transmitted to susceptible animals through a biting midge vector (*Culicoides* spp.). Disease severity can range from subclinical to lethal depending on many factors, including the serotype of the virus and the species of ruminant infected. BT disease has long been recognized as a disease of agricultural animals; however, it was not until the early 20th century that BTV was described as the causative agent of the disease [1]. Since the initial description, BTV has been recognized to be endemic in most countries around the world between the latitudes 35° S and 40° N [2]. Due to this expansive range and the nature of segmented RNA viruses, many serotypes have emerged throughout the world, with over 29 serotypes currently described [3]. The emergence of a novel serotype in a region can have devastating consequences on local agricultural and wildlife ruminant species. In 2006, BTV8, typically endemic to sub-Saharan Africa, emerged in northern Europe and caused immense damage to the agricultural industry both in terms of animal life and monetarily [4]. Interestingly, some of the European countries impacted by the BTV8 emergence were outside the typical BTV zone, suggesting that climate change may be expanding the biting midge host range [5]. More recently, the emergence of BTV3 in the Netherlands, which was originally detected in September 2023, has been shown to be a much more severe outbreak compared to the 2006 emergence [6]. Currently, the impacted area has expanded to many European countries, including Germany, Belgium, France, Luxembourg, and Denmark, and is expected to spread to the UK. There are no treatments for BTV disease, and a current marketed vaccine offers coverage for 3–5 serotypes and thus is not extensively used globally. However, Europe is testing a quadrivalent that includes BTV3 to mitigate the current outbreak [6,7]. Given the gravity of potential novel outbreaks in both agricultural and wildlife populations, it is considered a notifiable disease by the WOAH.

It is known that the viral immune response is critical to combating BTV and plays a major role in determining the severity and lethality of the disease [8]. We previously characterized the temporal viral pathogenesis and immunological response to BTV17 in sheep [9]. We observed a robust CD8^+^ T-cell response with an induction of proinflammatory cytokines (CXCL10 and IFNγ) just prior to the expansion of the CD8^+^ T-cell population. However, BT disease pathogenesis can vary, depending on the vertebrate host species it infects and the serotype of BTV associated with infection [5,10]. To better understand the impacts of these two variables, we evaluated cohorts of sheep and Reeves’ muntjac deer to understand differences in the native host species’ response to two BTV serotypes, BTV10 and BTV17. Both species have been shown to be impacted by BTV infection [11] Given that the spread of BTV is dependent on the *Culicoides* biting midge vector, we also evaluated the efficiency of BTV uptake and replication in midges over the longitudinal course of BTV infection. Building on our previous studies evaluating BTV17 in sheep, this study evaluates a different endemic BTV serotype, BTV10, in sheep as well as testing both BTV10 and BTV17 in a separate susceptible host species, the muntjac deer. In our studies, all the animals were successfully infected with BTV, yet BTV10 and BTV17 infections were less severe, and the immune response was more subdued in muntjac deer despite similar trends in proinflammatory cytokine and CD8^+^ T-cell responses in both muntjac deer and sheep. Midges successfully acquired BTV infections from a blood meal taken from infected sheep, but not muntjac deer. Our findings fill gaps in our understanding of the immunological responses and impacts of BTV10 and BTV17 infections in native agricultural and wildlife hosts, providing insights into the natural spread of these viruses in nature.

## 2. Materials and Methods

### 2.1. Animal Care and Husbandry and Clinical Signs of Disease

Six female Rambouillet sheep (*Ovis aries*), ranging from 3 to 8 years of age, were used in this experimental study. The range in age of sheep used for these studies was due to the necessity for stringent conditions to determine cohort inclusion. Prior to the start of the study, ewes were screened for current and/or historic exposure to BTV by RT-qPCR and serology. The ewes were also screened for pregnancy to further reduce confounding variables to the study. Female animals were chosen for housing purposes. During inoculation, blood collections, and midge feedings, the sheep were manually restrained for the procedure. The thirteen Reeves’ muntjac (*Muntiacus reevesi*) deer were bred and housed within CSU facilities. Due to the challenge of maintaining the colony of muntjac, animals were used as available. The animals’ age ranged from 1 to 15 years and they were a mix of males and females. The sheep cohort sizes were determined by the following considerations: several BTV serotypes are endemic to the Northern Colorado area and thorough testing was performed on the candidate sheep to ensure that (1) none were currently infected with BTV, (2) they were all naive to any previous BTV exposures, and (3) to verify none were pregnant. Female sheep were only used in this study due to the complexity of housing and maintaining male rams. Given these strict inclusion criteria, the available experimental sheep were placed in cohorts to ensure sufficient negative and positive animals for the study. Muntjac deer were bred in-house to provide animals for experimentation. As with the sheep, the muntjac deer cohorts were maximized to ensure sufficient negative and positively infected animals despite this constraint. For the BTV inoculation and sample collection, the muntjac were anesthetized intramuscular with Butorphanol (0.45 mg/kg), Azaperone (0.35 mg/kg), and Medetomidine (0.08 mg/kg), and were monitored during recovery. At the conclusion of the study, all the cohorts of animals were anesthetized prior to euthanasia with euthanasia consisting of an intravenous injection of pentobarbital sodium with phenytoin (1 mL per 4.5 kg).

At the start of the study (Day 0), the animals were subcutaneously inoculated with 1 mL media (mock-inoculated) or BTV17 or BTV10 (at a titer of 1.4 × 10^6^ TCID_50_ mL^−1^). The media used for mock-inoculated animals was identical to the cell culture media used to propagate the BTV isolates used in this study. The animals were monitored daily for BTV clinical signs throughout the experiment: elevated body temperature; respiratory distress; behavioral changes (apathy, lethargy); nasal/eye discharge; ulcers in the eye or nasal cavity; excessive salivation; and/or facial edema. The animals were humanly euthanized at the end of the study. All the animals were handled in strict accordance with the guidelines for animal care and use provided by the United States Department of Agriculture (USDA), National Institutes of Health (NIH), and the Association for Assessment and Accreditation of Laboratory Animal Care International (AAALAC), and all animal work was approved by Colorado State University Institutional Animal Care and Use Committee (IACUC #1400). Blood collections entailed blood and serum samples taken from the jugular vein at regular intervals.

### 2.2. Culicoides Sonorensis Handling and Processing

*C. sonerensis* midges were acquired from the Arthropod-Borne Animal Diseases Research Unit (United States Department of Agriculture, Agricultural Research Service, Manhattan, KS, USA), specifically from the AK colony that originated from the field in Owyhee Co., Idaho, August 1973 [12]. *C. sonorensis* were shipped overnight by air at one day of age and held at 27 °C and provided with 10% (*w*/*v*) sugar water ad libitum for two days before the infection studies commenced [13,14]). Access to sugar water was removed 24 h prior to blood feeding. To feed on the vertebrate hosts, the *C. sonorensis* were kept in paper containers of non-treated paper topped with sheer pantyhose over the lid for access to feeding and air exchange and pressed against the sheered back (sheep) or belly (muntjac) of the host animal. Separate groups of *C. sonorensis* fed on all the experimental animals every 7 days of the study. After receiving the bloodmeal, the *C. sonorensis* groups were chilled for four minutes at −20 °C and placed on a modified chill table to facilitate the sorting and collection of engorged females from each infection group into containers to be housed at 27 °C. Immediately after feeding (F0), pools of 5 blood-fed females from each infection group were collected and evaluated for uptake of BTV via pan BTV qRT-PCR. The remaining *C. sonorensis* were maintained at 27 °C with 10% (*w*/*v*) sugar water ad libitum to 10 days to allow the expansion of midge BTV infection to occur. After 10 days (F10), the surviving midges were pooled into groups of 5 and accessed for BTV by RT-qPCR, as was the F0 group. Humidity and temperatures of incubators were monitored daily.

### 2.3. BTV Virus Isolate and Propagation

The BTV (order *Reovirales*, family *Sedoreoviridae*, genus *Orbivirus*, species bluetongue virus) serotypes used in this study, BTV10 and BTV17, are endemic to the United States and have been described in previous studies by our group [9,15,16]. Use of the virus was limited to one passage post expansion. The BTV17 (USA1988/CA, also known as BTV-17-CA) isolate is a characterized field isolate from California (GenBank, MT952971-MT952980). Briefly, it was isolated from whole blood harvested from a naturally occurring BTV infection in a clinically affected sheep. BTV10 California 1952 (BTV-10) (Bluetongue virus, type 10, strain 8, ATCC^®^ VR-187™) was procured from ATCC and passaged eight times on BHK 21 cells, as previously described [15]. Infectious titers for BTV10 and BTV17 were determined by a 50% tissue culture infectious dose (TCID_50_) in triplicate with negative controls and calculated using the Reed–Muench method in the BHK 21 cell line. BHK 21 cells were propagated in BHK 21 media comprising EMEM, 10% fetal bovine serum (certified, heat-inactivated, US origin from Sigma Aldrich, St. Louis, MO, USA), 10% tryptose phosphate broth (Sigma Aldrich), and 1% penicillin streptomycin (10,000 U/mL). The cells were incubated at 37 °C with 5% CO_2_ and passaged every three to four days when culture flasks were 80–90% confluent.

### 2.4. BTV Competitive ELISA

Sera collected from all the experimental animals were tested for anti-BTV antibodies using a BTV-specific VP7 cELISA kit (Veterinary Medical Research and Diagnostics, Bluetongue Virus Antibody Test Kit, Inc., Pullman, WA, USA) and performed according to the instructions of the supplier. The positivity threshold was set at 60% inhibition. BTV was not detected in mock-inoculated controls at any time point.

### 2.5. Longitudinal PCR Viral Detection in Blood and Terminal Tissues

**Figure 1:**
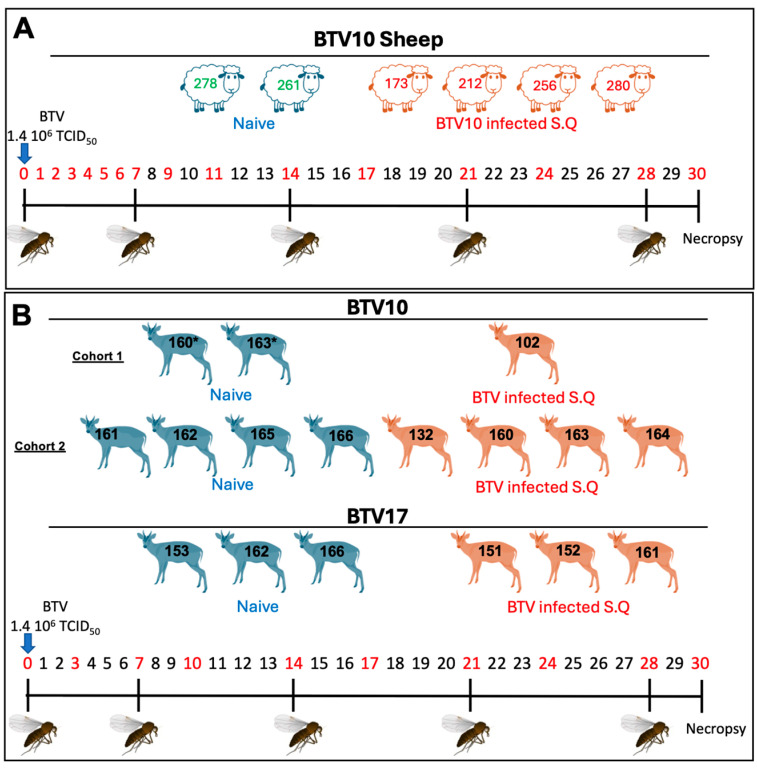
Experimental study design. (**A**) Sheep and (**B**) muntjac deer are shown by representative drawings. Negative animals (shown in blue) and positive animals (shown in red) are divided into cohorts. Individual animal numbers were assigned for each experimental animal and indicated by a numerical overlay for each representative animal. At D0, animals were inoculated with BTV. The days denoted in red are those when blood draws occurred. The midge icon represents days on which naive midges were fed on the experimental animals. Animals with (*) after their number denote those used as negatives in the BTV10 study (non-terminal), which were subsequently incorporated as positively infected animals in a second experimental cohort.

Whole blood (EDTA) was collected from each animal at regular intervals (Figure 1) throughout the study. Detection of BTV viral RNA was performed as previously described [17,18]. Viral genomic material was isolated using the MagMAX Pathogen RNA/DNA kit (Applied Biosystems, Foster City, CA, USA) per the manufacturer’s instructions. BTV-specific primers (BTV-Fwd:5′-TGGAYAAAGCRATGTCAAA -3′, BTV Rev: 5′-ACRTCATCACGAAACGCTTC-3′, BTV-Probe 5′/56-FAM/ARGCTGCAT/ZEN/TCGCATCGTACGC/3IABkFQ/3′) were used in an RT-qPCR reaction using SuperScript™ III Platinum One-step RT-qPCR kit (Invitrogen, Carlsbad, CA, USA). Samples were considered positive if the cycle to threshold (Ct) value was below 37. For midge preparation, prior to the nucleic acid extraction, *C. sonorensis* were homogenized in a volume of 50 µL EMEM per *C. sonorensis* (i.e., 250 µL for pools of five *C. sonorensis*) [19] and evaluated by RT-qPCR as described above.

Viral genomic detection from terminal tissues (lung, spleen, muscle, retropharyngeal lymph node, liver, and kidney) was performed by Trizol phenol-chloroform isolation, and cDNA was generated using a Roche Transcriptor First strand cDNA synthesis kit (as per the kit instructions) and the RT-qPCR was performed using the same primers and methodology.

### 2.6. Flow Cytometry, Complete Blood Counts, and Blood Chemistry

The following anti-sheep antibodies were used according to the manufacturer’s specifications: CD8α (clone ST8, Cat# SHP2002, WSU, Pullman, WA, USA), CD4 (clone S-17D, Cat #S-GT2002, WSU), CD72 (clone 2-104) [20], FITC conjugated anti-mouse (Cat# 1071-02, Southern Biotech, Birmingham, AL, USA), BV421 conjugated anti-mouse (Cat# 115-675-075, Jackson, Myrtle Beach, SC, USA), and PE conjugated anti-mouse (Cat# Ab970024, Abcam, Cambridge, UK). EDTA-Blood collected from sheep and muntjac was cleared of RBCs by a red blood cell lysing buffer (ammonium chloride) for 3 min at room temperature and washed repeatedly with PBS. The RBC cleared cells were incubated with the corresponding antibodies followed by incubation with a secondary antibody-conjugated fluorophores for 30 min to 1 h at room temperature and washed with PBS. The samples were fixed in 2% paraformaldehyde for 5 min, washed and resuspended in PBS. The samples were passed through a 35-μm cell strainer (Corning Life Sciences, Tewksbury, MA, USA) immediately before analysis on an Aurora spectro-cytometer (Cytek, Becton Dickinson, Franklin Lakes, NJ, USA). The data were analyzed using FlowJo (v.10) software.

Aliquots of EDTA-blood were submitted to the CSU Veterinary Diagnostic Laboratory for complete blood counts and blood chemistry. The analysis was sent as a report and data were collated by the researchers.

### 2.7. Cytokine Array

The cytokine array was performed on serum collected at indicated timepoints as per the manufacturer’s guidelines (Raybiotech, Cat# QAO-CYT-1, Peachtree Corners, GA, USA). Efficacy of use on muntjac serum was performed on blood from a negative animal that was stimulated (25 ng/mL PMA and 1 μg/mL ionomycin) in vitro prior to BTV experimentation. Fluorescent data scanning was performed by Raybiotech scanning services and raw data were provided for analysis and data collation. The serum cytokine biomarkers analyzed include CXCL10, IFNγ, and TNFα. Each sample was performed in at least triplicate (with four detection replicates per well). Concentrations were determined by a standard curve provided and performed alongside experimental arrays.

### 2.8. Statistical Analysis

The student’s *t*-test and two-way ANOVA were used to calculate the significance for comparison of two matched groups and three or more unmatched groups, respectively, using Prism 10 (GraphPad, San Diego, CA, USA). The results were considered statistically significant at a *p*-value of less than 0.05.

## 3. Results

### 3.1. Experimental Study Design

To build upon our previous study [9] to determine how BTV10 and BTV17 impact BT disease pathogenesis and immune response in native host species, we implemented an experimental design that mirrors our previous studies of BTV17 infection in sheep (Figure 1). We established cohorts of confirmed BTV negative sheep and in-house bred Reeves’ muntjac deer for this study. The cohorts of animals were inoculated subcutaneously in the nape of the neck at day 0 (0 DPI) with a dose of 1.4 × 10^6^ TCID_50_ mL^−1^ of BTV10 or BTV17. The animals were monitored daily, and peripheral blood was collected daily for the first week then at every three days (sheep) or twice weekly (muntjac). At 7-day intervals, confirmed BTV negative midges (~250/feeding) were permitted to blood-feed on sheep or deer and were further monitored and processed to determine their ability to uptake BTV. At the conclusion of the study, at 4 weeks post-inoculation, all the animals were euthanized and necropsied, and relevant tissues were collected for further analysis.

### 3.2. Detection of Circulating BTV and Humoral Response in Peripheral Blood of Experimental Animals

**Figure 2:**
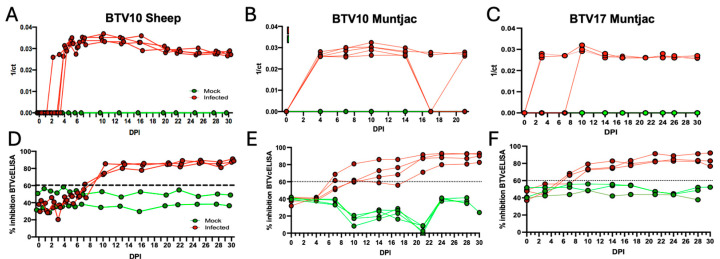
Experimentally inoculated sheep and muntjac deer exhibited detectable viremia during the experimental time course. (**A**) Detection of BTV10 genomes by RT-qPCR in sheep and (**B**) muntjac deer. (**C**) Detection of BTV17 genomes by RT-qPCR in muntjac. Peripheral blood was drawn and processed for BTV detection. BTV was not detected in mock-inoculated controls at any time point. Assay used a threshold of 0.04 and the controls were valid. The data are represented as 1 over the Cycle threshold (Ct). Serum taken at blood draw time points was evaluated for detection of anti-BTV antibodies using a BTV-specific competitive ELISA (cELISA). (**D**) BTV10 in sheep, (**E**) BTV10 in muntjac, and (**F**) BTV17 in muntjac. A sample was considered positive if it was above 60% inhibition of the signal threshold.

To determine if the inoculated animals had detectable viral genomes over the course of the study, we evaluated peripheral blood by RT-qPCR using BTV-specific primers. We found circulating BTV10 in sheep peripheral blood as early as 2 DPI (Figure 2A). All the positively inoculated sheep revealed robust detection in peripheral blood by 4 DPI that remained detectable throughout the remainder of the study. These findings of BTV10 RNA detection in sheep are highly consistent with previous findings observed with BTV17 RNA in experimentally infected sheep [9]. We then sought to determine if viral circulation would be significantly altered in a different susceptible species, muntjac deer. In the muntjac cohort, inoculated with BTV10, we found that all the positively inoculated animals had detectable circulating BTV by 4 DPI, similar to what we detected in sheep BTV10 infections (Figure 2B). Interestingly, we detected less consistent viral signals in BTV10-inoculated muntjac by 17 DPI with two animals having no circulating BTV10 for the remainder of the study. Given that BTV10 infections in sheep remained robust throughout the study and faltered in the muntjac, we next sought to determine the temporal profile of BTV17 detection in muntjac deer (Figure 2C). As was observed in sheep, circulating BTV17 was first detected as early as 2 DPI, and as late as 10 DPI. Unlike BTV10 in the muntjac, all BTV17-inoculated muntjac remained positive until the end of the study. All the negative control cohorts remained free of viral infection at all time points. In summary, sheep appear to have highly consistent infection with both BTV10 and BTV17, whereas muntjacs have a more robust infection with BTV17 as compared to BTV10.

To determine if BTV10- and 17-inoculated animals mounted a humoral response, we performed a BTV-specific competitive ELISA (cELISA). Samples were considered positive if they crossed the 60% inhibition threshold. A productive anti-BTV humoral response was established by all BTV10-infected sheep by 10 DPI and was maintained until the termination of the study (Figure 2D). Similarly, BTV10-infected muntjac mounted a robust response with half of the inoculated animals crossing the positivity threshold by 7 DPI, one additional muntjac at 14 DPI, and the last muntjac by 21 DPI. All the muntjac remained above the positivity threshold for the remainder of the study. BTV17-infected muntjac exhibited a unique response (Figure 2F), a slightly accelerated response by 7 DPI (2/3 muntjac) (Figure 2E), and 10 DPI (1/3 muntjac), which was retained through the remainder of the study.

### 3.3. Detection of BTV Deposition in Terminal Tissues of Experimental Animals

**Figure 3:**
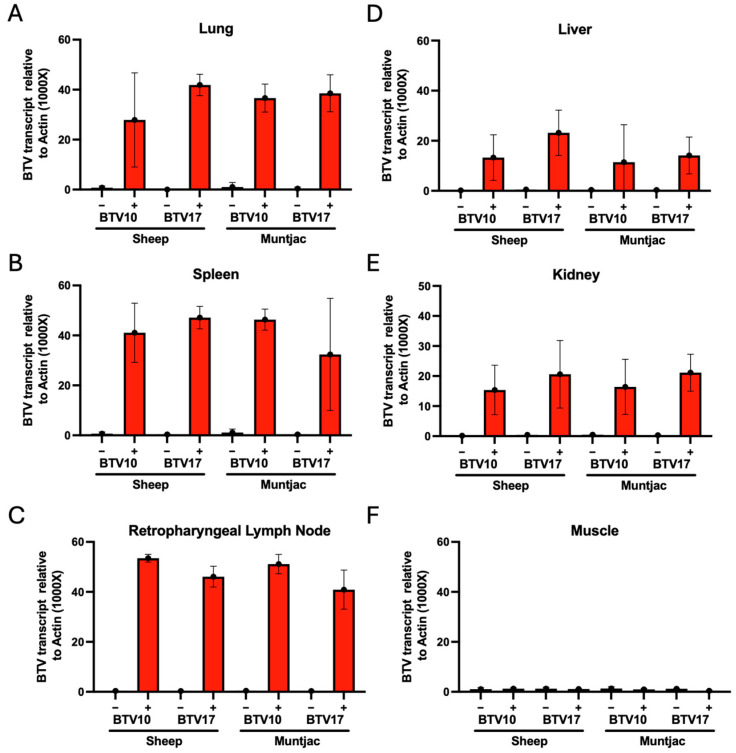
Evaluation of terminal tissues for BTV10 or BTV17 by RT-qPCR using serotype-specific primers. Lung (**A**), spleen (**B**), retropharyngeal lymph node (**C**), liver (**D**), kidney (**E**), and muscle (**F**) were evaluated for BTV viral genome burden.

To determine the extent of peripheralization of the BTV infection in experimentally inoculated animals, we evaluated a battery of terminal tissues collected at the conclusion of the study. The tissues were evaluated for BTV10 or BTV17 RNA using RT-qPCR and standardized to the vertebrate host housekeeping gene action to determine the relative quantity of viral RNA. The tissues evaluated include lung, spleen, retropharyngeal lymph node, muscle (collected from the rear thigh of the animal), kidney, and liver. These tissues were selected as lung, spleen, and lymph node are known to harbor BTV in infected animals [11]. Alternatively, liver, kidney, and muscle are not traditionally known to harbor large quantities of BTV [21]. Upon evaluation, we found a robust detection of BTV10 and BTV17 RNA in the respective cohorts of sheep and muntjac lungs (Figure 3A), spleen (Figure 3B), and lymph node (Figure 3C). This is in agreement with our previous study and with the current literature [9,21]. Analysis of sheep cohort tissues revealed less RNA detection in liver and kidney than lung and lymphoid tissues, yet RNA levels in all tissues were substantial (Figure 3D). RNA levels in muntjac liver and kidneys were less than those detected in sheep; nevertheless, they were also robust. Lastly, detection of BTV in the muscle of all the experimental cohorts showed minimal (Ct greater than 35) to no detection (Figure 3F). These data suggest that while muscle tissue does not harbor BTV RNA during infections, lung and primary lymphoid tissues, including spleen and lymph nodes, as well as tissues historically not recognized to harbor BTV RNA (liver and kidney), contain substantial burdens by 30 DPI.

### 3.4. Clinical Signs of BTV

**Figure 4:**
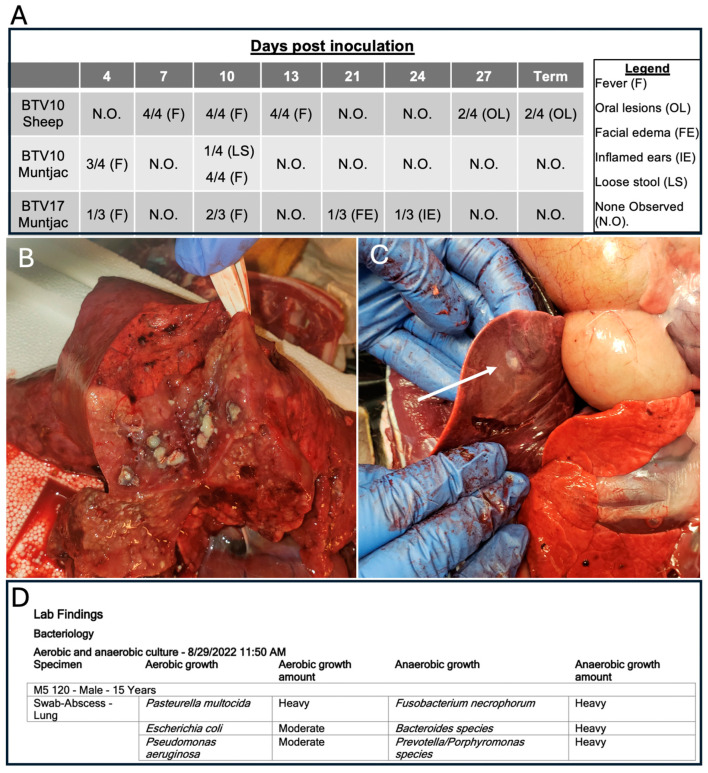
Table of clinical signs of 3 experimentally infected BTV cohorts (**A**). Aged BTV10 muntjac terminal collections. Images of gross anatomical infection of (**B**) lung and (**C**) liver. White arrow indicates liver abscess (**D**) Bacteriology results of samples submitted to CSU diagnostics.

All the animals were monitored for clinical signs of BT disease throughout the longitudinal course of the study. Interestingly, unlike the robust clinical presentation we previously identified in sheep infected with BTV17, sheep infected with BTV10 exhibited more subtle signs of disease (Figure 4A). BTV10-inoculated sheep presented with elevated temperatures as compared to their mock-infected counterparts from 7–13 DPI. Oral lesions were observed in this cohort at 27 DPI, which were confirmed at necropsy. The muntjac cohorts infected with BTV17 and BTV10 also demonstrated slightly elevated temperatures at 4 and 10 DPI, irrespective of the serotype. Except for a single BTV10-inoculated older muntjac that succumbed to infection, minimal clinical signs were observed. Both BTV-inoculated muntjac cohorts exhibited minor signs of clinical disease. A single BTV17-inoculated muntjac presented with facial edema at 21 DPI, and inflamed ears at 24 DPI, and a single BTV10-inoculated muntjac had loose stool at 10 DPI.

Interestingly, despite the observation of less robust signs of BTV infection in muntjac than in sheep, a single BTV10-inoculated muntjac demonstrated an unusual disease course. Unique to the muntjac cohort, this male was considerably older (15 yrs) than others in the cohort (1–10 yrs). At 17 DPI, minor clinical signs of elevated temperature, labored breathing, salivation, coughing, anorexia, and behavioral depression were noted. Palliative care, including antibiotics and fluids, was provided by attending veterinarians. The animal showed a marked improvement until 24 DPI when symptoms returned and became more pronounced. The decision was made to euthanize the muntjac. Upon necropsy, the lungs and liver (Figure 4B,C) appeared to be burdened by bacterial infections and thus samples were submitted for bacterial culturing. The results confirmed secondary bacterial infections, including growth of populations of common oral and respiratory microbiome organisms including *E. coli*, *Prevotella*, and *Bacteroides* species (Figure 4D), suggesting an opportunistic expansion of these species. More pathogenic bacterial populations were identified including *P. aeruginosa* and *F. necrophorum*. The former are associated with opportunistic infections in immune compromised individuals [22], and the later with necrotic laryngitis and liver abscesses in cattle [23]. Taken together, these data suggest that the aged animal was unable to control infection and ultimately succumbed to a secondary bacterial infection, as has been suggested previously [21,24]. Yet across the muntjac cohort, serology demonstrating exposure to BTV and the detection of peripheral blood RNA viral genomes remained similar for all the muntjac (i.e., no differences were revealed between the older muntjac that presented with clinical disease and the younger ones that did not present with clinical disease).

### 3.5. Immune Changes in BTV-Inoculated Sheep and Muntjac

**Figure 5:**
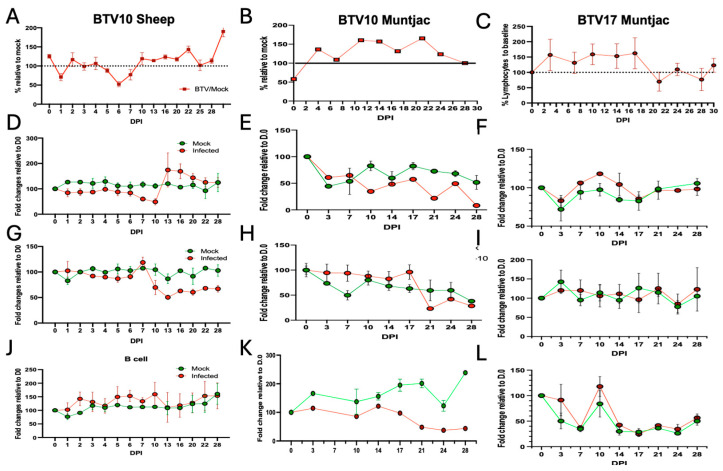
Experimentally infected sheep and muntjac exhibited dynamic immune responses over the study time course. Peripheral blood was analyzed for specific cell subsets using flow cytometry and cell-specific antibodies. The data are presented with (left column) BTV10 sheep, (middle column) BTV10 in muntjac, and (right column) BTV17 in muntjac. Broader cell populations, as indicated by complete blood counts, show lymphocytes (**A**–**C**). CBC data are shown as BTV-infected relative to mock (set to 100%) for each timepoint. Specific cell populations were identified by flow cytometry (**D**–**F**) CD8^+^ T cells, (**G**–**I**) CD4^+^ T cells, (**J**–**L**) B cells were detected and evaluated. The data are shown as mock-infected (green) and BTV-infected (red). To account for animal-to-animal variation, all data points were standardized to signal at day 0.

As we have previously observed dynamic changes within the lymphocyte populations of immune cells in response to BTV17 infection in sheep [9], we sought to evaluate these immune populations in our current BTV-inoculated cohorts. In the BTV10-inoculated sheep, we found similar modulation in total lymphocyte populations as was previously observed in the BTV17-infected sheep (Figure 5A). This included a dramatic decrease in the lymphocyte population at 1 DPI, which recovered by 3 DPI, only to decline again at 6 DPI, before recovering and expanding beyond baseline. As the lymphocyte response was consistent between both BTV10 and BTV17 serotypes in sheep, we next wanted to determine how the lymphocyte population in muntjac deer respond to BTV10 and BTV17. The temporal lymphocyte population modulation in the BTV10 muntjac cohort revealed similar kinetics, albeit more subdued, to that observed for the BTV10 and BTV17 sheep cohorts (Figure 5B). In the BTV10-inoculated muntjac, the lymphocyte population expanded by 3 DPI with a decrease in cell population at 7 DPI and 17 DPI before expanding to a peak at 21 DPI and returning to baseline by the end of the study. In the BTV17-inoculated muntjac, we observed an elevated lymphocyte population that was observed 2 DPI and was maintained until 16 DPI, when there was a sudden decline in the population (Figure 5C). The lymphocyte population recovered at 24 DPI and remained close to baseline for the remainder of the study.

To evaluate specific subsets, we employed flow cytometry using validated antibodies against specific cell populations. In the BTV10-inoculated sheep, we saw a decline in CD8^+^ T cells through 10 DPI, which expanded above mock levels by 13 DPI and slowly returned to mock levels by the end of the study (30 DPI) (Figure 5D). The muntjac CD8^+^ T-cell responses also declined by 10 DPI but lacked the rebound to mock levels noted in the sheep cohort (Figure 5E). In the BTV17-inoculated muntjac, higher percentages/total CD8^+^ T cells were demonstrated between 7 to 14 DPI that ultimately returned to the mock-inoculated levels by 17 DPI and remained at these levels until the end of the study (Figure 5F). Together, these data show a consistent CD8^+^ T-cell response in sheep and muntjac revealing a differing response between the two serotypes. The other major lymphocyte population implicated in the anti-BTV response is the CD4^+^ T-cell population. Upon evaluation, we found CD4^+^ T cells in the BTV10-infected sheep exhibited highly consistent trends to those observed in the BTV17-inoculated sheep [9] (Figure 5G), i.e., a modest expansion at 7 DPI, which declined throughout the course of the study. In BTV10-inoculated muntjac, a consistent level of CD4^+^ T cells were demonstrated until 17 DPI, which was followed by a slight expansion in this cell population until 28 DPI (Figure 5H), after which there was a decline in the CD4^+^ T-cell population, which was maintained at below mock levels until the end of the study. Interestingly, muntjac inoculated with BTV17 showed no dynamic changes in CD4^+^ T cells throughout the entire study (Figure 5I). Lastly, B-cell detection in BTV10-inoculated sheep (Figure 5J) revealed modest changes when compared to their mock-inoculated counterparts. In contrast, BTV17-inoculated muntjac (Figure 5K) showed a dramatic reduction in B cells throughout the entirety of the study. The B-cell percentage detected in the BTV10-inoculated muntjac (Figure 5L) was very similar to BTV10-infected sheep, where no differences from the mock were observed. Taken together, these data demonstrate that the immune response in sheep to both serotypes is highly consistent, and that the immune response in muntjac is more varied and much more subtle when compared to the responses in sheep cohorts.

### 3.6. Cytokine Changes in BTV-Infected Sheep and Muntjac

**Figure 6:**
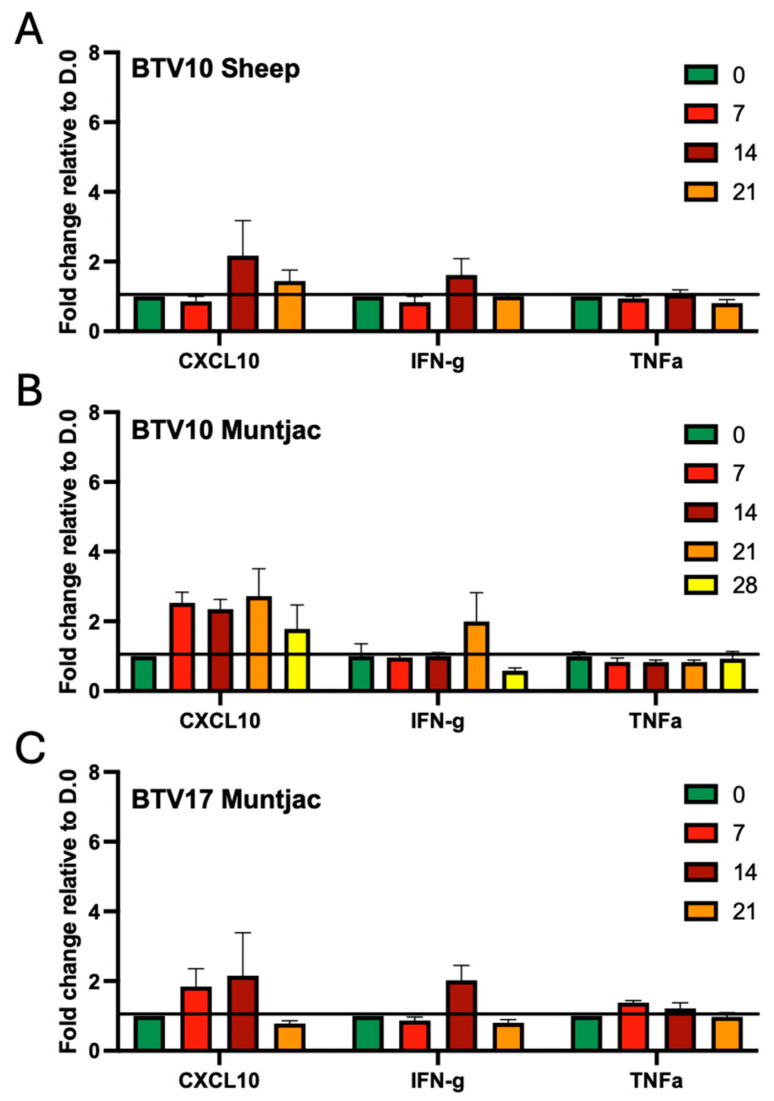
Cytokine changes during BTV infection experimental animals. Serum cytokines CXCL10, IFNγ, and TNFα, were evaluated from the peripheral blood experimental animals at day 0, 3, 7, 14, 21, and 28 using the Raybiotech cytokine array. (**A**) BTV10 in sheep, (**B**) BTV10 in muntjac, and (**C**) BTV17 in muntjac. The data are shown as RFU with the background signal removed.

Cytokines help drive the immune response. We have previously observed that prior to the CD8^+^ T-cell expansion in BTV17-infected sheep, there is an increase in the proinflammatory cytokines CXCL10 and IFNγ [9]. To determine how these factors respond in our experimental cohorts, we evaluated the cytokine response using a cytokine array. In the BTV10-inoculated sheep, CXCL10 and IFNγ increased at 14 DPI while TNFα remained at roughly baseline throughout the study (Figure 6A). Although the response is a bit subdued and slightly more delayed than what was observed in the BTV17-inoculated sheep, the trends remain consistent. Interestingly, we found that BTV10- and BTV17-inoculated muntjac showed a robust induction of CXCL10 at 7 DPI, which remained elevated until 28 DPI and 14 DPI, respectively (Figure 6B,C). Although both serotypes induced IFNγ, BTV10 produced peak levels at 21 DPI, whereas BTV17 peaked at 14 DPI. Levels returned to below baseline in both cohorts by the following collection, suggesting a swift and short-lived response. TNFα levels in both muntjac cohorts were modest, if any change was noted, which is consistent with what was observed in both sheep cohorts.

### 3.7. Blood Chemistry Changes in BTV-Infected Sheep and Muntjac

**Figure 7:**
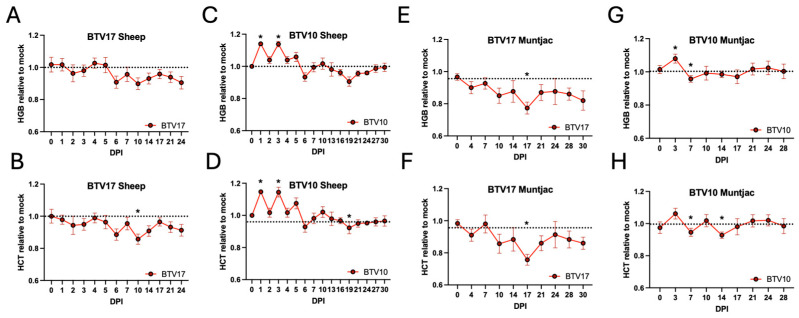
Blood chemistry was performed on peripheral blood taken at each time point. The data are shown as the average of BTV-inoculated animals relative to the average mock-infected animals. Hemoglobin (top) and hematocrit (bottom) of (**A**,**B**) BTV17 sheep, (**C**,**D**) BTV10 sheep (**E**,**F**) BTV17 in muntjac, and (**G**,**H**) BTV10 in muntjac. To account for animal-to-animal variation, all data points were standardized to signal at day 0. * indicate statistical significance with a *p*-value of less than 0.05.

In addition to the immune cell and cytokine analysis, we also evaluated blood chemistry panels for all the animals. Interestingly, we found that factors related to RBCs, hemoglobin and hematocrit, were significantly changed between the BTV serotypes in sheep and muntjac cohorts. We have included chemistry panel findings here from our previous BTV17-sheep infection study, as they were included in our published findings [9]. Marked reductions were seen in hemoglobin and hematocrit levels in the BTV17-inoculated sheep from 2, 6, and 10 DPI, which remained below mock levels until the termination of the study (Figure 7A,B). In contrast, BTV10-inoculated sheep exhibited significant spikes for both hemoglobin and hematocrit at 1 and 3 DPI, which declined at 6 and 19 DPI before recovering, and remained similar to mock controls for the remainder of the study (Figure 7C,D). Similar trends were seen in the BTV17 muntjac cohort as were observed in the BTV17 sheep (Figure 7E,F), where the hemoglobin and hematocrit were reduced by 4 DPI and remained below mock levels for the remainder of the study. Interestingly, muntjacs inoculated with BTV10 showed a brief spike, as was observed in the BTV10-infected sheep at 3 DPI, and had minor dips at 7 and 14 DPI, but then remained very consistent with the mock levels for the remainder the study (Figure 7G,H). Given that BTV has an affinity for RBCs, it has been speculated that BTV attachment to RBCs allows it to persist in the host [25]. These host species and serotype-related differences may be suggestive of distinct differences in BTV pathogenesis.

### 3.8. C. sonorensis Feeding on Infected Vertebrate Hosts

To assess when during infection circulating BTV can be acquired via the insect vector, *C. sonorensis* midges were fed on BTV- or mock-inoculated sheep and muntjac once a week until the end of the study period. Fed midges were split into two cohorts: cohort (1) was immediately evaluated for the presence of BTV RNA by RT-qPCR (F0), and cohort (2) was incubated at 27 °C for ten days prior to assessment for BTV RNA by RT-qPCR (F10). The incubation period (cohort 2) is representative of the time interval needed for BTV to infect *C. sonorensis* and subsequently transmit the infection during the next vertebrate blood meal (also referred to as extrinsic incubation period, or EIP). Pools of midges were used to enhance the detection robustness, as has previously been performed [15] (Table 1). Midges that fed on mock-inoculated animals remained negative.

In the midges that were fed on the BTV10-inoculated sheep, BTV10 was detected in every viable pool of midges (F0) that fed on the sheep at 7 and 14 DPI. Furthermore, we found that *C. sonorensis* midge pools that fed on sheep at 7 DPI remained positive for BTV RNA 10 days after feeding (F10). However, despite all F0 midge pools feeding on sheep at 14 DPI, and testing BTV positive at F0, none of the midge pools were positive at F10. For the remainder of the study, only one single F0 midge pool at the terminal collection was BTV positive. These data suggest that *C. sonorensis* midges acquire and transmit the BTV10 agent during the early stages of vertebrate infection.

We also evaluated *C. sonorensis* midge ability to acquire BTV17 infection from experimentally inoculated sheep using samples collected and stored from our previously published study, which used the same study design [9]. We found that successful midge feedings at F0 had intermittent BTV RNA positivity at each timepoint evaluated (D7, D14, D21, Terminal). Interestingly, despite detection at each F0 timepoint, we were not able to detect positivity in any of the midges at 10 days post feeding (F10). The detection of BTV17 in midges at each timepoint suggests that BTV17 from infected sheep may be taken up by midges over a longer period during the vertebrate infection.

As we have observed species-to-specific differences in BTV viral detection in sheep and muntjac, we also wanted to determine if midge feeding and positivity differed between the two species and viral serotypes. Analysis of *C. sonorensis* midge pools that fed on BTV10- and 17-inoculated muntjac revealed very little positivity throughout the study. Although sparce, BTV RNA of both serotypes was detected in F0 midge pools that fed on BTV-inoculated muntjac at 7 (BTV10, #163, and BTV17, #161) and 21 DPI (BTV17, #161). Interestingly, midges that fed on the muntjac that succumbed to infection revealed no BTV detection throughout the study time course. These data suggest that BTV propagation and transmission in the native cervid host may be more of an obstacle than in the sheep livestock host.

## 4. Discussion

The negative impacts of emerging bluetongue disease in ruminants were clearly demonstrated in 2006 following the emergence of BTV8 in northern Europe, resulting in widespread economic and agricultural losses [4]. The ongoing impacts of climate change increase the potential for continued BTV reassortment and serotype emergence that will result in similar devastating outcomes. This includes a novel emergence in the United States, which is heavily invested in cattle, ovine, and other ruminant products [26]. To mitigate these impacts, a greater understanding of the disease across serotypes and host species is necessary to develop therapeutic targets. We have previously evaluated the immunological and pathogenic response to BTV17 in sheep [9]. Here, we have evaluated sheep (a major agricultural species in the US) and Reeves’ muntjac deer (a related cervid species to many wildlife species in the US) using two serotypes endemic to the US (BTV10 and BTV17). We found that across both viral serotypes and animal host species, all the positively inoculated animals showed detectable circulating virus during a longitudinal time course (30 days) (Figure 2). Interestingly, differences were seen in the rate of initial detection and duration of infection between both species and serotypes investigated. Most notably, while BTV10 infections in sheep were robust and demonstrated circulating virus in all animals by 4 DPI, little detectable signal was noted in BTV10-inoculated muntjac, and only near the end of the study. This suggests a more transient BTV10 infection in the muntjac species. In comparison, BTV17 infections in muntjac and sheep were equally robust and durable throughout the study course. Taken together, these data suggest that sheep are more susceptible to BTV infections of either serotype and, while muntjac show susceptibility, their infections may be less severe, barring additional complications. The susceptibility of infection is emphasized by the evaluation of the terminal tissues showing that lungs and primary lymphoid tissues (spleen and lymph node) harbor the most viral genomes, liver and kidney show decent viral genome detection, and muscle show little evidence of infection detection at all at the end of this 30-day study (Figure 3).

Similarly, all the BTV-inoculated animals (sheep and muntjac) mounted a serological response that crossed the 60% inhibition threshold for the BTV cELISA. Interestingly, muntjac BTV10 infections resulted in a slightly delayed antibody responses as compared to the other experimental cohorts in several animals, with the last crossing the threshold at 17 DPI. In conjunction with the detection of circulating virus, this may suggest that BTV10 requires a longer incubation period in muntjac, resulting in a delayed serological response.

Immunologically, the responses to BTV infection varied most dramatically by species rather than serotypes (Figure 5 and Figure 6). We have demonstrated that sheep inoculated with BTV10, as compared to our previous study where we evaluated sheep inoculated with BTV17, showed remarkably consistent changes in the immune response, with some interesting changes in both the timing and intensity of this response. Trends in both the CD4^+^ and CD8^+^ T cells were highly consistent between BTV10 and BTV17 sheep. Interestingly, the timing of the CXCL10 response in the BTV10-inoculated sheep was delayed by several days as compared to those inoculated with BTV17, suggesting that it may assist the T-cell response rather than drive a CD8^+^ T-cell expansion, as we previously had hypothesized. Although the cytokine profile trends for both serotypes and species evaluated in this study were consistent, immune cell modulations seen in muntjac were more varied. CD8^+^ T-cell expansion early in disease of the BTV17-inoculated muntjac suggests a role for CD8s in controlling the infection. Of interest, muntjac inoculated with BTV10 had a reduced detection of BTV RNA near the end of the study and established a reduced level of CD8^+^ T cells for the majority of the study. This cohort did, however, show uniquely that an increase in CD4 T cells early in infection may help play a role in controlling the disease, as has been recently suggested [27]. The majority of BTV10-inoculated muntjac presented with minor clinical signs of BT disease. Yet one aged muntjac succumbed to secondary infection potentially caused by the previously described immune suppression driven by BTV [21,24]. This case may reflect that mature and vital wildlife ruminants are able to successfully mount an immune response to stave off major complications of BT disease, while advanced age and poor health/secondary infections may result in more severe disease and poorer outcomes. It has been reported that BTV is a major cause of hemorrhagic disease and mortality in white-tailed deer in several states in the U.S. [28].

Evaluation of the blood chemistry of the infected animals reveals a highly diverse response in red blood cell related factors, hemoglobin and hematocrit (Figure 7). Although we observed this trend in the previous BTV17 in sheep study [9], we were hesitant to report on these findings due to repeated blood draws and the impact blood loss alone may have on these factors. However, when we observed similar unique findings in the cohorts of this study, and that the negatives were drawn at the same timepoints creating an internal control, we determined this may have important implications for characterizing BT disease. Given the highly consistent responses to both serotypes in sheep, it was unexpected to see such dramatic differences in the RBC factors. Follow-up studies are needed to evaluate BTV burdens in sheep to determine potential differences between serotype ability to bind to RBCs, as has been previously observed [25]. The serotype-specific changes observed in BTV17-inoculated sheep are further supported by the consistent changes observed in the BTV17-inoculated muntjac. This may represent a mechanism utilized by BTV17 that is not as robustly initiated by other serotypes, including BTV10.

We found that feeding *C. sonorensis* on the positively infected animals revealed some interesting trends. The midges that fed on the sheep infected with both BTV serotypes showed a greater ability to uptake BTV. Interestingly, *C. sonorensis* that fed on the BTV10-infected sheep at 7 DPI showed robust positivity that was maintained to 10 days post midge feeding. Although BTV RNA was detected in fed midges at 14 DPI (F0), the continuation of the infection was not observed in the midge (F10). We observed that this is roughly the time at which a significant immune response to BTV was observed in the vertebrate host. This may suggest that the optimal time for a midge acquiring the virus through a blood-feed is very early in the vertebrate infection, and that when the host establishes an anti-BTV response, the window of infection begins to close. The sparse infection rates noted in *C. sonorensis* that fed on muntjac are curious as the infection rates in both mammalian host were quite similar. This suggests that there may be a difference in BTV transmission efficacy between livestock and wildlife ruminant species. Further studies are needed to further define BTV transmission dynamics for each species. Overall, these studies demonstrate the variability of infection of BTV serotypes in different host species. We observed that sheep species had a much more defined disease and immune response that then was able to be transmitted to the midge vector. In contrast, the cervid species had a more subdued infection and immune response, which was less likely to be passed on to the midge vector. However, it was in the cervid species that we observed the only moribund infection. Taken together, the ecology of BTV transmission and disease status across species is highly variable and combating this disease will likely require serotype- and host species-specific considerations to mitigate endemic and potential emerging infections.

## Figures and Tables

**Table 1:** Results of midge feeding on all experimental cohorts. D represents DPI for vertebrate hosts. F represents day of midge feeding; F0 is day of feeding and F10 is 10 days post feeding of the midges. Red numbers indicate positivity in the group. Animals with (*) after their number denote those used as negatives in the BTV10 study (non-terminal), which were subsequently incorporated as positively infected animals in a second experimental cohort. Numbers indicate unique animal identifier. Red animals numbers were inoculated with BTV and green animals numbers were mock inoculated animals. Numbers (#) indicate unique animal identifier. Red animals numbers were inoculated with BTV and green animals numbers were mock inoculated animals.

	Animal #	D7.F0	D7.F10	D14.F0	D14F.10	D21.F0	D21.F.10	Term.F0	Term.F10
**BTV17 Sheep**	215	1/1	0/0	2/2	0/1	1/2	0/2	0/2	0/0
	257	0/0	0/0	0/0	0/0	0/2	0/0	1/2	0/1
	221	0/0	0/0	1/2	0/0	1/2	0/1	0/2	0/0
	237	0/0	0/0	0/2	0/0	0/2	0/0	1/2	0/0
	226	0/0	0/0	0/0	0/0	0/2	0/3	0/2	0/2
	33	0/0	0/0	0/0	0/0	0/3	0/1	0/0	0/0
	17	0/0	0/0	0/0	0/0	1/3	0/0	0/0	0/0
	10	0/0	0/0	0/0	0/0	0/3	0/1	0/0	0/0
	69	0/2	0/1	0/2	0/1	0/2	0/1	0/1	0/0
	252	0/2	0/0	0/2	0/1	0/2	0/0	0/2	0/1
	28	0/0	0/0	0/0	0/0	0/3	0/1	0/0	0/0
	9	0/0	0/0	0/0	0/0	0/3	0/1	0/0	0/0
	7	0/0	0/0	0/0	0/0	0/3	0/1	0/0	0/0
**BTV10 Sheep**	280	3/3	3/3	0/0	0/0	0/3	0/0	0/3	0/0
	256	3/3	0/0	1/1	0/0	0/3	0/0	1/3	0/0
	212	3/3	0/0	3/3	0/1	0/3	0/3	0/3	0/2
	173	3/3	1/1	3/3	0/1	0/3	0/0	0/3	0/0
	278	0/3	0/3	0/0	0/0	0/3	0/0	0/3	0/0
	261	0/3	0/0	0/3	0/0	0/3	0/2	0/3	0/2
**BTV17 Muntjac**	151	0/3	1/1	0/3	0/0	0/3	0/0	0/3	0/0
	152	0/3	0/1	0/3	0/1	0/3	0/1	0/3	0/2
	161	1/3	0/1	0/3	0/0	1/3	0/0	0/3	0/0
	153	0/3	0/1	0/3	0/1	0/1	0/0	0/1	0/1
	162	0/1	0/1	0/3	0/1	0/4	0/2	0/2	0/2
	166	0/3	0/2	0/0	0/0	0/1	0/1	0/3	0/0
**BTV10 Muntjac**	102	0/1	0/1	0/1	0/0	0/1	0/0	0/1	0/0
	132	0/1	0/3	0/1	0/0	0/0	0/1	0/1	0/1
	160	0/1	0/0	0/1	0/0	0/0	0/0	0/1	0/0
	163	1/1	0/1	0/1	0/0	0/1	0/0	1/1	0/0
	164	0/3	0/0	0/0	0/0	0/0	0/1	0/0	0/0
	160 *	0/1	0/1	0/1	0/0	0/1	0/0	0/1	0/0
	163 *	0/1	0/0	0/1	0/0	0/1	0/0	0/1	0/0
	161	0/1	0/1	0/0	0/0	0/1	0/0	0/1	0/1
	162	0/1	0/1	0/1	0/0	0/0	0/1	0/1	0/1
	165	0/1	0/1	0/1	0/0	0/1	0/0	0/1	0/0
	166	0/0	0/0	0/2	0/0	0/0	0/1	0/0	0/1

## Data Availability

Dataset available upon request from the authors.

## References

[1] Acevedo A.M., Hinojosa Y., Relova D., Perera C.L. (2016). Bluetongue virus: A known virus, a current threat. Rev. Salud Anim..

[2] Mahy B.W., Van Regenmortel M.H. (2008). Encyclopedia of Virology.

[3] Jiménez-Cabello L., Utrilla-Trigo S., Calvo-Pinilla E., Moreno S., Nogales A., Ortego J., Marín-López A. (2020). Viral vector vaccines against bluetongue virus. Microorganisms.

[4] Meiswinkel R., Baldet T., De Deken R., Takken W., Delécolle J.C., Mellor P.S. (2008). The 2006 outbreak of bluetongue in northern Europe—The entomological perspective. Prev. Vet. Med..

[5] El Moustaid F., Thornton Z., Slamani H., Ryan S.J., Johnson L.R. (2021). Predicting temperature-dependent transmission suitability of bluetongue virus in livestock. Parasites Vectors.

[6] Santman-Berends I.M.G.A., van den Brink K.M.J.A., Dijkstra E., van Schaik G., Spierenburg M.A.H., van den Brom R. (2024). The impact of the bluetongue serotype 3 outbreak on sheep and goat mortality in the Netherlands in 2023. Prev. Vet. Med..

[7] Ranjan K., Prasad M., Brar B., Lambe U., Kumar R., Ghosh M., Prasad G. (2019). Bluetongue virus vaccine: Conventional to modern approach. Acta Virol..

[8] Rodríguez-Martín D., Louloudes-Lázaro A., Avia M., Martín V., Rojas J.M., Sevilla N. (2021). The interplay between bluetongue virus infections and adaptive immunity. Viruses.

[9] Westrich J.A., McNulty E.E., Carpenter M., Burton M., Reed K., Nalls A., Sandoval A., Mayo C., Mathiason C.K. (2023). Monitoring longitudinal immunological responses to bluetongue virus 17 in experimentally infected sheep. Virus Res..

[10] Caporale M., Di Gialleonorado L., Janowicz A., Wilkie G., Shaw A., Savini G., Van Rijn P.A., Mertens P., Di Ventura M., Palmarini M. (2014). Virus and host factors affecting the clinical outcome of bluetongue virus infection. J. Virol..

[11] Maclachlan N.J., Drew C.P., Darpel K.E., Worwa G. (2009). The pathology and pathogenesis of bluetongue. J. Comp. Pathol..

[12] Jones R.H., Foster N.M. (1978). Relevance of laboratory colonies of the vector in arbovirus research—*Culicoides variipennis* and bluetongue. Am. J. Trop. Med. Hyg..

[13] Bonneau K.R., Mullens B.A., MacLachlan N.J. (2001). Occurrence of genetic drift and founder effect during quasispecies evolution of the VP2 and NS3/NS3A genes of bluetongue virus upon passage between sheep, cattle, and *Culicoides sonorensis*. J. Virol..

[14] Bonneau K.R., DeMaula C.D., Mullens B.A., MacLachlan N.J. (2002). Duration of viraemia infectious to *Culicoides sonorensis* in bluetongue virus-infected cattle and sheep. Vet. Microbiol..

[15] Carpenter M., Kopanke J., Lee J., Rodgers C., Reed K., Sherman T.J., Graham B., Cohnstaedt L.W., Wilson W.C., Stenglein M. (2024). Evaluating Temperature Effects on Bluetongue Virus Serotype 10 and 17 Coinfection in *Culicoides sonorensis*. Int. J. Mol. Sci..

[16] Kopanke J.H., Lee J.S., Stenglein M.D., Mayo C.E. (2020). The genetic diversification of a single bluetongue virus strain using an in vitro model of alternating-host transmission. Viruses.

[17] Ortega J., Crossley B., Dechant J.E., Drew C.P., MacLachlan N.J. (2010). Fatal bluetongue virus infection in an alpaca (*Vicugna pacos*) in California. J. Vet. Diagn. Investig..

[18] Hofmann M.A., Renzullo S., Mader M., Chaignat V., Worwa G., Thuer B. (2008). Genetic characterization of toggenburg orbivirus, a new bluetongue virus, from goats, Switzerland. Emerg. Infect. Dis..

[19] Kopanke J., Lee J., Stenglein M., Carpenter M., Cohnstaedt L.W., Wilson W.C., Mayo C. (2021). Exposure of *Culicoides sonorensis* to enzootic strains of bluetongue virus demonstrates temperature-and virus-specific effects on virogenesis. Viruses.

[20] Young A.J., Marston W.L., Dessing M., Dudler L., Hein W.R. (1997). Distinct recirculating and non-recirculating B-lymphocyte pools in the peripheral blood are defined by coordinated expression of CD21 and L-selectin. Blood J. Am. Soc. Hematol..

[21] Parsonson I.M. (1990). Pathology and pathogenesis of bluetongue infections. Bluetongue Viruses.

[22] Bodey G.P., Bolivar R., Fainstein V., Jadeja L. (1983). Infections caused by Pseudomonas aeruginosa. Rev. Infect. Dis..

[23] Tadepalli S., Narayanan S.K., Stewart G.C., Chengappa M.M., Nagaraja T.G. (2009). Fusobacterium necrophorum: A ruminal bacterium that invades liver to cause abscesses in cattle. Anaerobe.

[24] Saminathan M., Singh K.P., Maity M., Vineetha S., Manjunathareddy G.B., Dhama K., Malik Y.S., Ramakrishnan M.A., Misri J., Gupta V.K. (2021). Pathological and immunological characterization of bluetongue virus serotype 1 infection in type I interferons blocked immunocompetent adult mice. J. Adv. Res..

[25] Brewer A.W., MacLachlan N.J. (1994). The pathogenesis of bluetongue virus infection of bovine blood cells in vitro: Ultrastructural characterization. Arch. Virol..

[26] Gibbs E.P.J., Tabachnick W.J., Holt T.J., Stallknecht D.E. (2008). US concerns over bluetongue. Sci. -New York Then Wash..

[27] Newbrook K., Khan N., Fisher A., Chong K., Gubbins S., Davies W.C., Sanders C., Busquets M.G., Cooke L., Corla A. (2024). Specific T-cell subsets have a role in anti-viral immunity and pathogenesis but not viral dynamics or onwards vector transmission of an important livestock arbovirus. Front. Immunol..

[28] Kring E.K., Stallknecht D.E., D’Angelo G.J., Kohl M.T., Bahnson C., Cleveland C.A., Salvador L.C., Ruder M.G. (2024). Patterns of Hemorrhagic Disease in White-Tailed Deer (*Odocoileus virginianus*) in the Great Plains of the USA 2024, 1982–2020. J. Wildl. Dis..

